# Rare Ring Chromosome [r(15)]: Cytogenetic Abnormality in TP53-Mutated De Novo AML-M4 Masked as Gastrointestinal Bleed With Rapidly Progressing Hyperleukocytosis and Leukostasis

**DOI:** 10.7759/cureus.46119

**Published:** 2023-09-28

**Authors:** Ifeoma Kwentoh, Maria Lorraine Bugayong, Rahman Olusoji, Tasheka McPherson, Meena Ahluwalia

**Affiliations:** 1 Medicine, Columbia University, New York, USA; 2 Internal Medicine, Columbia University at Harlem Hospital Center, New York, USA; 3 Pulmonology, Columbia University at Harlem Hospital Center, New York, USA; 4 Oncology, Columbia University at Harlem Hospital Center, New York, USA

**Keywords:** rare cytogenetic anomalies, gastrointestinal bleeding (gib), nup98 gene • aml • rare mutation • chromosome 11p15 • acute myeloid leukemia, ring chromosome 15, human genetics and epigenetics, myelodysplastic (mds)/myeloproliferative neoplasm (mpn) disease spectrum, aml gene mutations

## Abstract

TP53-mutated (TP53m) acute myeloid leukemia (AML) comprises only 5-15% of de novo AML, associated with poor survival outcomes due to its resistance to conventional therapy. Ring chromosomes, an even more rare subset of genetic anomalies, occur in only 2% of cases. We report a unique case of de novo AML with both TP53 and ring chromosome anomalies leading to a catastrophic outcome in a 72-year-old male who initially presented with gastrointestinal bleeding (GIB) and urethral stone status post-cystoscopy with J-stent placement. He had no history of chemotherapy use, radiation, benzene exposure, or any other risk factors except for his age. He was noted to have pancytopenia, for which bone marrow biopsy, flow cytometry, and cytogenetic studies were done. Biopsy reported an interesting next-generation sequenced TP53-mutated AML, which correlates with a low rate of response to standard chemotherapy except for bone marrow transplants. Notably, with a complex aberration of 45 XY with multiple translocations (t), deletions (del), inversions (inv), derivative (der) breakpoints, aneuploidy, and rare ring and maker chromosomes, his case was complicated with rapid-onset and very severe hyperleucostasis, reflecting the prognostic value of this rare cytogenetic configuration. The patient expired within 48 hours of diagnosis, despite the urgent initiation of cytoreductive therapy and the mitigation of tumor lysis syndrome with Rasburicase. To the best of our knowledge, this is one of the first AML-M4 patients with rapid-onset leucostasis and the demise of next-generation sequences (NGS) in a de Novo AML patient with this rare complex combination.

## Introduction

Acute myeloid leukemia (AML) is a neoplastic proliferation of clonal myeloid cells that are truncated in their growth and associated with grave outcomes [1,2]. AML has the potential to occur de novo or following certain risk factors such as radiation exposure, chemotherapeutic agents, and benzene [3,4]. This malignant disorder may also occur from myeloproliferative neoplastic transformation [5]. M4-AML, a class of myeloid leukemias, almost always involves significant bleeding, bruising, petechiae, and infection [6,7]. Patients may have a very high leukocyte count and leukostasis syndrome, with varying presentations including central nervous system manifestations, hypoxia, and diffuse infiltrates on chest X-rays [8]. The cytogenetic and molecular classification of AML is mutually exclusive in diagnosis and targeted therapy [9,10]. Traditionally, AML is managed with hypomethylating agents (HMA), intensive chemotherapy (IC), and also venetoclax (VEN) combined with HMA (VEN + HMA) as an option [10]. Decitabine and azacitidine may also be combined with targeted therapies (FLT3 inhibitors, IDH inhibitors) [11].

Patients are usually expected to at least survive months, and palliative team discussion for hospice care would be considered at the time of diagnosis. A systematic review and meta-analysis was carried out to compare treatment outcomes in newly diagnosed, treatment-naïve patients with TP53-mutated (TP53m) AML, and despite improved responses seen with IC and VEN + HMA compared to HMA, survival endpoints remained poor with very limited health benefits cut across all treatment groups, including treatment-naïve patients [12]. This sheds more light on the demand for further research aimed at improving treatment for this difficult-to-treat population [12]. The association of AML with cytogenetic dyscrasias is a common occurrence, many of which are characterized by chromosomal abnormalities [13-15]. Cytogenetic aberrancy reflects reciprocal chromosomal translocation, which often leads to fusion genes that encode chimeric proteins [15,16].

Cytogenetic abnormalities involve either complete, partial loss, or gain of a chromosome; however, they play a significant role in the diagnostics, classification, prognostic value, and choice of therapy in AML [17]. The presence of very complex aberrations is associated with poor prognosis [17,18]. Many bodies of evidence have shown that TP53m are unique in functionality and form from the typical AML cases and have shown inconsistent responses to therapy [18].

## Case presentation

**Figure 1:**
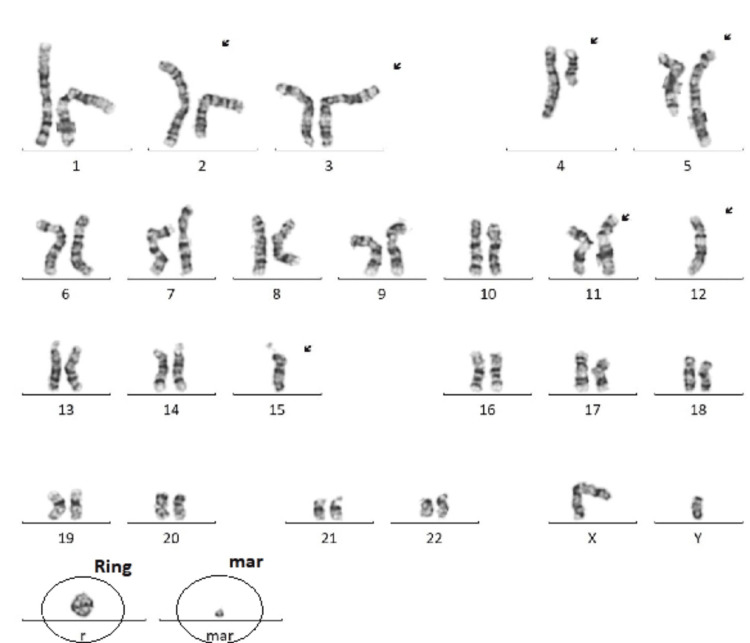
R: G-G banding show a rare ring and marker non-specific aberration for a particular FAB subtype. 45-4XYadd(2)(q13),der(3)t(1:3),(q21:p28),del(4)(q21;q35),der(5)ins(5;?)(q13:?),t(2:5)(q31:q33),der (11),t(11:15)(q14:q15)-12-15+r+0~1mar(13)/45-46. idem,-der (11;15),+der(11)t(11:14)(q14;q13),-14,+15[cp7]. FAB: French-American-British classification of acute myeloid leukemia. Simplified FAB classification of AML: M0 – undifferentiated; M1 –myeloblastic without maturation; M2 – myeloblastic with maturation (commonest type); M3 – promyelocytic; M4 – myelomonocytic (naegeli type).

**Figure 2:**
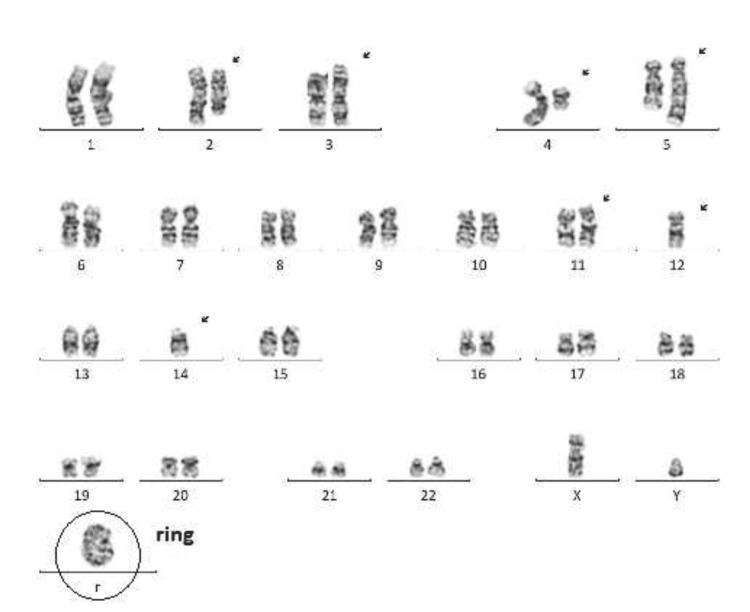
L: G-G banding show a rare ring chromosome 45,XY,add(2)(q13),der(3)t(1;3)(q2;p25),del(4)(q21q35),der(5)ins(5:?)t(2;5)(q31;q33),der(11)t(11:14)(q14;q13),-12,-14,+r.

A 74-year-old man with a history of Benign prostatic hyperplasia, type 2 diabetes, and nephrolithiasis presented with a three-day history of bright red blood per rectum and an infected wound on the right hand. Initial workup showed pancytopenia (hemoglobin 10; white blood count [WBC] 3.6; platelet 63) with no evidence of hepatic or splenic disease. He was started on empiric antibiotics for cellulitis. While awaiting endoluminal evaluation, he developed severe right-sided flank pain and an oliguric acute kidney injury (creatinine 6 uptrend from 0.8), concerning obstructive uropathy. An emergent cystoscopy did not show obstruction or signs of infection. Postoperatively, he developed acute hypoxic respiratory failure requiring mechanical ventilation and a medical intensive care unit, septic shock, and anuric renal failure requiring renal replacement therapy. Despite broad-spectrum antibiotics, he had persistent leukocytosis (WBC 33k). Peripheral smears showed metamyelocytes, warranting a bone marrow biopsy and flow cytometry. Results showed acute myeloid leukemia with a TP53 mutation, complex aberrations including multiple translocations (t), deletions (del), inversions (inv), derivatives (der), breakpoints, aneuploidy, and a rare ring chromosome 15 (Figures 1-2 and Table 1). Figures 1-2 all depict the cytogenetic analysis of our patient, which demonstrated cytogenetic analysis with an abnormal clone (13 out of 20 cells) characterized by complex aberrations and written as a composite karyotype. Numerical aberrations include the loss of one copy of chromosomes 12 and 15. Structural aberrations include the addition of material of unknown origin to 2q13, a derivative chromosome 3 resulting from an unbalanced translocation between chromosomes 1q21 and 3p25, interstitial deletion of chromosome 4q, a derivative chromosome 5 resulting from the insertion of unknown material into 5q13, a derivative chromosome 11 resulting from an unbalanced translocation between chromosomes 11q14 and 15q15, and the presence of a ring and a marker chromosome. Other clonal aberrations identified in a subset of clonal cells (7 out of 20 cells) include a derivative chromosome 11 resulting from an unbalanced translocation between chromosomes 11q14 and 14q13. Normal cells were never found in our patient, indicative of abnormal male karyotype aberrations.

These current cytogenetic results are compatible with a myeloid malignancy such as AML or possibly myelodysplastic syndrome (MDS). The aberrations identified are not specific to a particular AML French-American-British classification (FAB) subtype, with clinical and hemopathology correlations suggested. Furthermore, G-G banding shows a rare ring and marker nonspecific aberration for a particular FAB subtype. G-G banding shows a rare ring chromosome (Figure 2).

**Table 1:** Cytogenic analysis

Metaphases counted	Metaphases analyzed	Metaphases karyotyped	G-T-G band level	Culture types
20	20	12	400	24hrU

Within 48 hours of diagnosis, he developed rapid-onset leukostasis and hyperleukocytosis (WBC 110-120k). Despite initiating urgent cytoreductive therapy with hydroxyurea and prophylactic treatment for tumor lysis syndrome, the patient expired. Our patient had no history of radiation, benzene exposure, or any other risk factor except for age. His biopsy reported an interesting next-generation sequenced TP53-mutated AML with ring chromosome 15. To the best of our knowledge, this is one of the first AML-M4 with next-generation sequences (NGS) in de novo AML patients. Ring chromosome 15 [r (15)] Mosaic is a very rare genetic makeup requiring further research due to the diversity of both its phenotype and genotype, with further complexity in AML. Further analysis of the patient's bone marrow differentials showed 34% myeloblast with varying degrees of normoblast (22%; Table 2). CD117 was consistent with AML, non-acute promyelocytic leukemia (APL) type (Table 3).

**Table 2:** Bone marrow differentials of our patient

Cell type	Results	Normal range
Myeloblasts	34%	0–3%
Promyelocytes	4%	2–8%
Myelocytes	3%	10–13%
Metamyelocytes	2%	10–15%
Neutrophils/bands	14%	25–40%
Monocytes	5%	0–1%
Eosinophils	1%	1–3%
Basophils	<1%	0–1%
Lymphocytes	13%	10–15%
Plasma cells	1%	0–1%
Pronormoblast	1%	0–2%
Normoblasts	22%	15–25%

**Table 3:** Blast equivalent 34%, increased CD34(+) myeloblast (23%) consistent with AML, non-APL type. AML: acute myeloid leukemia, GIST: gastrointestinal stromal tumor, CD117 (c-KIT), APL: acute promyelocytic leukemia.

Antibody	Clone	Description	Results
CD34	QBEnd10	Endothelial and stem cells, GIST, BLAST	50% cells positive
CD117	YR145	c-kit ligand, myeloid and mast cell marker, GIST	50% cells positive

## Discussion

Cases of AML with cytogenetic dyscrasias, many of which are characterized by chromosomal abnormalities, have been reported [19]. They typically involve reciprocal chromosomal translocations, which often lead to fusion genes that encode a chimeric protein, while some involve complete or partial loss or gain of a chromosome. These cytogenetic abnormalities play a significant role in diagnostics, classification, prognostic value, and choice of therapy in AML. The TP53 mutation is associated with poor outcomes and high mortality [19,20]. Evidence has shown that TP53m are unique in functionality and form from the typical AML cases; hence, they exhibit inconsistent responses to therapy [19,20]. For these patients, a bone marrow transplant may be the only option. Ring chromosome 15 [r (15)] mosaic is a very rare genetic makeup with a diverse phenotype and genotype. Immunohistochemical analysis shows an increase in CD34+ CD117+ Myeloblast but zero Auer rods. Table 4 shows bone marrow flow cytometry analysis demonstrating B-cell-associated antibody types, their descriptions, and the percentage results of each type of cell composition in our patient.

**Table 4:** Ten color flow cytometry analysis for acute leukemia and myeloid/lymphoid neoplasm. CALLA: common acute lymphoblastic leukemia antigen, Ig: immunoglobulin, CLL: chronic lymphocytic leukemia.

Antibody	Description	Result
B-cell associated		
CD19	Pan B-cell antigen	0.55%
CD20	Pan B-cell antigen	0.55%
CD10	Follicle center B-cells, CALLA, myeloid subset	0.11%
CD11c	Monocytes, myeloid subset, hairy cell leukemia	13.67%
CD23	Mature B-cells, CLL	0.09%
CD25	Plan B-cell antigen	14.1%
CD71	Activated T and B-cells, hairy cell leukemia	0.29%
CD81	B-cells	0.58%
CD103	Activated cells, hairy cell leukemia	8.73%
CD200	B-Cells	1.25%
Kappa	Kappa Ig light chain, B-cells, plasma cells	0.32%
Lambda	Lambda Ig light chain, B-cells, plasma cells	0.17%

The patient's bone marrow iliac crest, core biopsy, clot, and aspirate smears showed AML of the non-APL type, best classified as acute myelomonocytic leukemia (AMML). The number of blasts (blast equivalents): 34% by cytomorphology; 50% by CD34 immunohistochemical (IHC) stain. The leukemia-associated phenotype (LAP) blasts show aberrant loss of human leucocyte antigen-DR (HLA-DR). His marrow cellularity was 90%, with marrow fibrosis absent (MF-0). Positive for the TP53 mutation (VAF 90%), per the next-generation sequence analysis of a recent blood sample collected on June 26, 2023. Iron storage increased: WBC 130 K/Ul, absolute neutrophil count (ANC) 24.26 K/Ul, absolute monocyte count 39,16 K/Ul (53.1%), hemoglobin 7.9 G/DL, platelet 34 K/Ul. Our patient's 10-color flow cytometry analysis for acute leukemia and myeloid/lymphoid neoplasm demonstrated hypercellular bone marrow with markedly increased immature and scattered foci of trilineage hematopoiesis. Phenotype of blasts: CD13+|CD33+ (moderate), CD64+|CD34+|CD38+|CD117+, and HLA-DR. Percentage of abnormal cells: 23% myeloblasts, cell size: large, Immature myeloid precursors positive for CD13| CD33|CD64 (dim), and CD117 are increased (23%) (myeloid-associated antibody) (Table 5). They are negative for HLA-DR. T-cell-associated antibody with CD2-CD57, with a description of the cell type and percentage results (Table 6: flow cytometry). Table 7 shows the blast/activation with leucocyte common antigen 82.32%.

**Table 5:** Myeloid-associated antibody NK cell: natural killer cell

Antibody	Description	Result
Myeloid associated		
CD11b	Mature myeloid cells, NK cells and T-Cell subset	27.72%
CD13	Myeloid cells, monocytes	97.64%
CD14	Mature monocytes	8.63%
CD15	Granulocytes	31.83%
CD16	Granulocytes, NK cells	19.7%
CD33	Myeloid cells, monocytes	40.68%
CD64	Monocytes	92.92%

**Table 6:** Flow cytometry and analysis of T-cell associated antibody. NK: natural killer.

Antibody	Description	Result
T-cell associated		
CD2	Thymic and peripheral T cells, NK cells	1.76%
CD3	Pan T-cell antigen, TCR-epsilon subunit	0.75%
CD4	Helper T-cells, thymocyte subset, monocytes	0.54%
CD5	Pan T-cells, mature B-cell subset (B1a cells)	1.08%
CD7	Thymic and peripheral T-cells, NK cells	0.84%
CD8	Suppressor T-cells, NK cells, thymocyte subset	0.37%
CD56	T-cell subset, NK cells	0.17%
CD57	T-cell subset, NK cells	0.26%

**Table 7:** Continuation of the flow cytometry and analysis of our patient. MHC-class II Ag: major histocompatibility complex-class II antigen.

Antibody	Description	Result
Blast/activation		
CD34	Precursor cells	17.14%
CD38	Activated T, B, myeloid cells, B-cell subset	0.15%
CD117	Stem cell receptor (c-kit), myeloid cells	9.28%
CD123	Anti-IL-3Ra, dendritic cell marker	2.28%
CD133	Precursor cells	3.18%
HLA-DR	MHC-class II Ag, B-cells, activated T-cells, monocytes	8.58%
Anchor		
CD45	Leukocyte common antigen	82.32%

Further research is needed to study driver mutations in AML with the TP53 mutation and chromosomal aneuploidies, deletions, derivatives, and translocation. Patients with chromatin-spliceosome and TP53-aneuploidy AML had poor outcomes overall, with the various class-defining mutations contributing independently and additively to the abysmal outcome. Ring chromosome 15 [r (15)] mosaic is a very rare genetic makeup requiring further research due to the diversity of both its phenotype and genotype, with further complexity in AML.

## Conclusions

Furthermore, large-scale cytogenetic studies are warranted in bigger population groups of AML patients in order to identify newly acquired chromosomal aberrations that may aid in cloning novel genes involved in the neoplastic process, with the ultimate goal of developing targeted therapeutic therapy. Research is needed to study these rare driver mutations in AML with TP53 mutations and chromosomal aneuploidies, deletions, derivatives, and translocation.
